# Predicting Renal Failure Progression in Chronic Kidney Disease Using Integrated Intelligent Fuzzy Expert System

**DOI:** 10.1155/2016/6080814

**Published:** 2016-02-02

**Authors:** Jamshid Norouzi, Ali Yadollahpour, Seyed Ahmad Mirbagheri, Mitra Mahdavi Mazdeh, Seyed Ahmad Hosseini

**Affiliations:** ^1^Department of Environmental and Energy, Islamic Azad University, Science and Research Branch, Tehran, Iran; ^2^Department of Medical Physics, School of Medicine, Ahvaz Jundishapur University of Medical Sciences, Ahvaz 61357-33118, Iran; ^3^Department of Civil and Environmental Engineering, K. N. Toosi University of Technology, Tehran, Iran; ^4^Iranian Tissue Bank & Research Center, Tehran University of Medical Sciences, Tehran, Iran; ^5^Nutrition and Metabolic Diseases Research Center, Ahvaz Jundishapur University of Medical Sciences, Ahvaz 61357-33118, Iran

## Abstract

*Background.* Chronic kidney disease (CKD) is a covert disease. Accurate prediction of CKD progression over time is necessary for reducing its costs and mortality rates. The present study proposes an adaptive neurofuzzy inference system (ANFIS) for predicting the renal failure timeframe of CKD based on real clinical data.* Methods.* This study used 10-year clinical records of newly diagnosed CKD patients. The threshold value of 15 cc/kg/min/1.73 m^2^ of glomerular filtration rate (GFR) was used as the marker of renal failure. A Takagi-Sugeno type ANFIS model was used to predict GFR values. Variables of age, sex, weight, underlying diseases, diastolic blood pressure, creatinine, calcium, phosphorus, uric acid, and GFR were initially selected for the predicting model.* Results.* Weight, diastolic blood pressure, diabetes mellitus as underlying disease, and current GFR_(*t*)_ showed significant correlation with GFRs and were selected as the inputs of model. The comparisons of the predicted values with the real data showed that the ANFIS model could accurately estimate GFR variations in all sequential periods (Normalized Mean Absolute Error lower than 5%).* Conclusions.* Despite the high uncertainties of human body and dynamic nature of CKD progression, our model can accurately predict the GFR variations at long future periods.

## 1. Introduction

Chronic kidney disease (CKD) is a growing health problem worldwide (about 10 to 15 percent of the adult population in USA, 11.2% in Australia, 10.1% in Singapore, 18.7% in Japan, and 8.3% to 18.9% in Iran) [1, 2]. It is a progressive disease associated with a high risk of cardiovascular diseases, mortality and morbidity rates, and high health care costs. Therefore, early detection of the disease to control and manage the consequences is of prime significance [3, 4]. The world health organization has estimated an amount of 1.1 trillion dollars as the cost of dialysis in the last decade [5]. Because of the dynamic nature of renal disease, its covert nature in the early stages, and heterogeneity of patients, predicting the renal failure progression with reasonable accuracy is necessary [6]. The renal failure progression can be considered as a function of various parameters including underlying renal diseases, blood pressure, hypertension, proteinuria, and age [7, 8].

In recent years, early diagnosis of the disease, especially determining the appropriate time to apply medical treatments for CKD has received great attention among clinicians and researchers [9–11]. Researchers through epidemiological and registry-based studies try to diagnose CKD in high risk patients as soon as possible and to control identified risk factors aggravating the disease progression to End Stage Renal Disease (ESRD) like hypertension, proteinuria, and hyperphosphatemia [9, 10, 12]. Based on the evaluations of these variables, different models have been developed to accurately predict progression to ESRD in CKD stages [9, 12]. However, they cannot quantitatively predict the variations of glomerular filtration rate (GFR) in the patients. Aguilar et al. evaluated the effective factors associated with CKD on 103 patients with mean age of 70.8 ± 13 years. The CKD associated factors were age >65 years old, male sex, positive history of cardiovascular disease, anemia, and obesity (BMI > 30). Their findings showed that age and anemia were the strongest factors associated with CKD in their population [13]. There have been several studies on the GFR variations among different populations of CKD patients [9, 10, 14–16]. In this regard, intelligent and machine learning methods have been increasingly used in the context of health and disease forecasting. Gaspari et al. (2004) compared renal function derived from 12 prediction equations with GFR measurement by plasma iohexol clearance as reference method in a group of 81 renal transplant recipients [16]. They concluded that all models overestimate renal function. However, Modification of Diet in Renal Disease (MDRD) and Walser formulas showed the best performance with the lowest bias and the highest precision [16]. Brier et al. (2003) compared artificial neural networks with traditional logistical regression in the prediction of delayed graft function (DGF) in kidney transplant patients. They compared the results of neural network with logistic regression method and founded more sensitivity of logistic regression in the prediction of no DGF (91 versus 70%), while the neural network was more sensitive to the prediction of eyes for DGF (56 versus 37%) [17]. Hussain et al. (2011) proposed an intelligent tool for detecting breast cancer using support vector machines (SVM). They compared the performance of the proposed method with other classification methods. Accordingly, SVM improved the diagnosis of the disease [18].

In recent years, fuzzy intelligent methods, especially fuzzy expert systems, have been increasingly used to predict different diseases. It seems that employing this method along with the clinical tools for diagnosis of different diseases and conditions may drastically reduce diagnostic errors. Fuzzy expert technique is more accurate than machine learning techniques.

Adaptive neurofuzzy inference system (ANFIS) is a learning based system based on the neural networks concepts. The ANFIS network used in the present study is based on the model proposed by Jang et al. [19]. This is a learner network equivalent to Takagi-Sugeno fuzzy inference system. Learning in this network is continuous update of the network parameters. Factors of layers I and IV are of learner type that, respectively, determine membership functions and first-order estimated function. The ANFIS training algorithm is a hybrid algorithm in which ordinary least squares algorithm is used to update coefficients of output functions (*f*_*i*_), while error back propagation algorithm is used to update fundamental factors of the system [19, 20].

If we can model and predict the renal function worsening, we can effectively manage this disorder. In this regard, an appropriate parameter should be considered as the marker of disease worsening. The GFR is the only reliable parameter of the renal function and progression of CKD [16]. The Cockroft-Gault (C-G), MDRD, and chronic kidney disease epidemiology collaboration (CKD-EPI) equations are the most common and validated equations to calculate GFR [21]. For different age and racial groups, these equations yield different accuracy in estimating renal function [21]. According to the clinical measurements (independent physiological parameters) and clinical outcomes, when GFR reaches less than 15 cc/kg/min/1.73 m^2^, renal replacement therapy (RRT) including dialysis or transplant is necessary for the patient survival. If GFR variation is predicted in a CKD patient, we can appropriately predict the time to reach GFR threshold value of 15 cc/kg/min/1.73 m^2^. In other words, the time for renal replacement therapy is predicted. In this study, speed of GFR decrease in CKD patients has been predicted based on the follow-up of the CKD patients during the study. To our knowledge, there has not been any efficient method proposed for predicting the timeframe of CKD worsening in individual cases [22]. Furthermore, fuzzy intelligent techniques have not yet been used to predict renal function worsening. The present study proposes an ANFIS based model for predicting the renal failure timeframe of CKD based on a 10-year real clinical data. The main objective is to provide a practical and reliable method with acceptable accuracy to find underpinning of a decision support system in health care and to support clinical decision making systems.

## 2. Materials and Methods

### 2.1. Data Collection

All the data of the present study were the clinical records of a cohort study of newly diagnosed CKD patients who were serially admitted to the Clinic of Nephrology, Imam Khomeini Hospital (Tehran, Iran), during October 2002–October 2011. The inclusion criteria for CKD definition included small sized kidney in ultrasound images or GFR less than 60 cc/kg/min/1.73 m^2^ for more than 3 months. The datasets were built using the clinical and laboratory data of different parameters. All the procedures of the study were approved by the ethics committee of Tehran University of Medical Sciences that completely coincide with the Declaration of Helsinki Ethical Principles for Medical Research Involving Human Subjects. The written consent form was obtained from each patient to participate in the study.

The patients were divided into two groups according to the pattern of their adherence to follow-up schedule in the clinic. A total of 465 CKD patients were enrolled in the study. The test group consisted of 389 patients who continuously (at least every six months) were visited in the clinic. The control group consisted of 76 patients who did not regularly follow their visit schedule in the clinic, but their visits had lasted at least one year. The details of demographic data and clinical measurements of the patients are presented in Table 1.

### 2.2. Input Selection

The present study used ANFIS neural networks to predict GFR values and compared the accuracy of the method. At each visit, patient's demographic data, weight, blood pressure, blood sample test variables including serum creatinine levels, fasting plasma glucose levels, lipid profile, calcium, phosphorus, hemoglobin, and other parameters were monitored. The appropriate treatments for blood pressure, bone mineral metabolism indices, and hemoglobin control were administered to each patient based on the clinical evaluations. The GFR was calculated using MDRD equation. The end point of data collecting for each patient was either GFR value less than 15 cc/kg/min/1.73 m^2^, start of RRT, or patient death. All quantitative variables were used as continuous to have a better training of model. According to the nephrologists' opinions and the kidney function, ten variables were initially selected as influencing parameters on the GFR variations. These variables included age, sex, weight, underlying diseases, diastolic blood pressure (dbp), creatinine (Cr), calcium (Ca), phosphorus (P), uric acid, and GFR. They were considered as the inputs of the predicting model. Some of these variables did not necessarily show strong correlation with the output (GFR_(*t*+*p*)_). On the other hand, in ANFIS model, input number reduction increased the accuracy of prediction and better training of model. Therefore, only the more significant inputs were selected according to the level of their correlation with the output. Pearson correlation coefficients test was used to determine the most significant input variables to be introduced in the ANFIS model. The Pearson correlation coefficients test was used because of the continuous nature of the variables.  Table 2 represents the correlation coefficients between the inputs and output (GFR_(*t*+*p*)_) at 6-month interval. Of the 10 inputs, only four inputs underlying disease, dbp, weight, and GFR_(*t*)_ have an acceptable correlation with the output (GFR_(*t*+*p*)_). Therefore, just these four variables were selected for future processing and modeling. In the next step, the GFR values were predicted at 6-, 12-, and 18-month intervals using ANFIS network model. The real data recorded during a ten-year period were recorded at 6-month intervals. Therefore, the GFR values were predicted for three sequential 6-month intervals at 6-, 12-, and 18-month intervals. The GFR_(*t*+*p*)_ with *p* = 1,2, 3 represents the GFR values at 6-, 12-, and 18-month intervals; that is, if *p* = 1, GFR prediction is performed for the subsequent 6-month interval.

### 2.3. Building Training and Test Datasets

The first step to train all neural networks and accurate modeling was to divide data into training and test datasets. Training data were used to optimize the weights and other parameters in the model. The test data were used to evaluate the quality of estimates and forecasts. In all further processing and modeling, the test dataset was not used for training models. Test data realistically simulated the model in the case where there was no information about the future.

The test data were randomly selected so that all data had an equal chance to participate in the selection process. The test datasets are usually selected among 30 to 40% of the available data. In this study, 30% of the data were selected as test dataset. The remaining 70% were used as training dataset to estimate and train models.

### 2.4. Fuzzification of Input Variables

Genfis3 code in MATLAB was used to fuzzify input variables and to establish the rule base. Genfis3 uses fuzzy *c*-means (FCM) clustering technique to fuzzify variables. The membership functions are Gaussian.

### 2.5. Establishing a Fuzzy Rule Base for ANFIS

**Figure 1:**
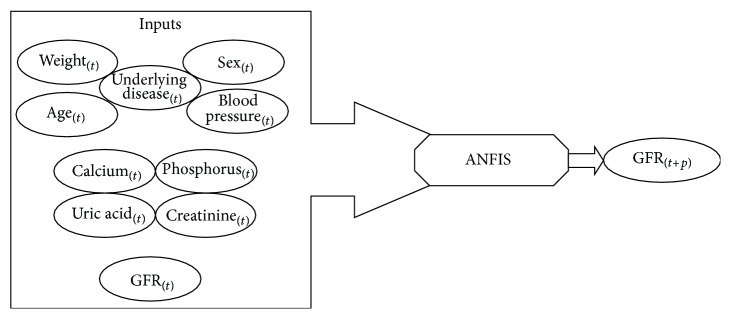
Schematic diagram of predicting model and input of variables.

**Figure 2:**
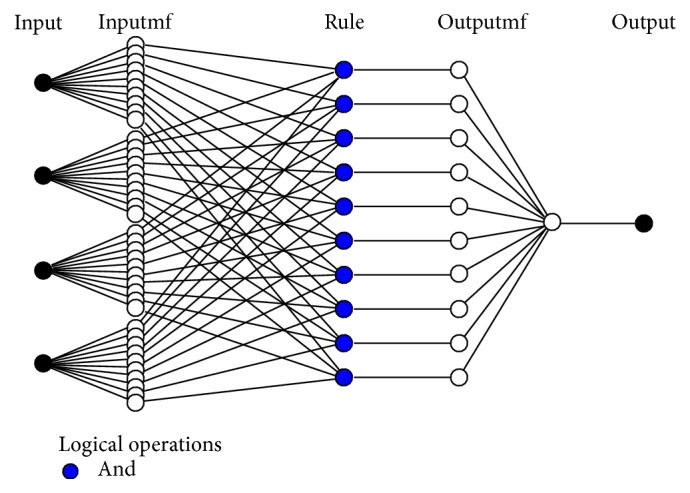
The ANFIS network structure used in the study to predict GFR values.

It is easy to establish a fuzzy rule base for ANFIS after fuzzifying variables using FCM clustering technique in Genfis3. The number of fuzzy rules is equal to the number of membership functions of input variables (11 functions). Thus, 11 fuzzy rules have been created in the rule base and used to estimate GFR values. Figures 1 and 2, respectively, show the ANFIS network structure and schematic diagram of the predicting model used in the present study.

## 3. Results

A total of 465 CKD patients were evaluated in the study, 277 of them were male. Diabetes mellitus was the underlying disease in 153 patients (33%). The GFR values ranged from 45 to 60 cc/kg/min/1.73 m^2^ in 154 patients (33.1%); 30–45 cc/kg/min/1.73 m^2^ in 215 (46.2%); and 15–30 cc/kg/min/1.73 m^2^ in 96 patients (20.7%).

### 3.1. GFR Prediction with ANFIS

**Figure 3:**
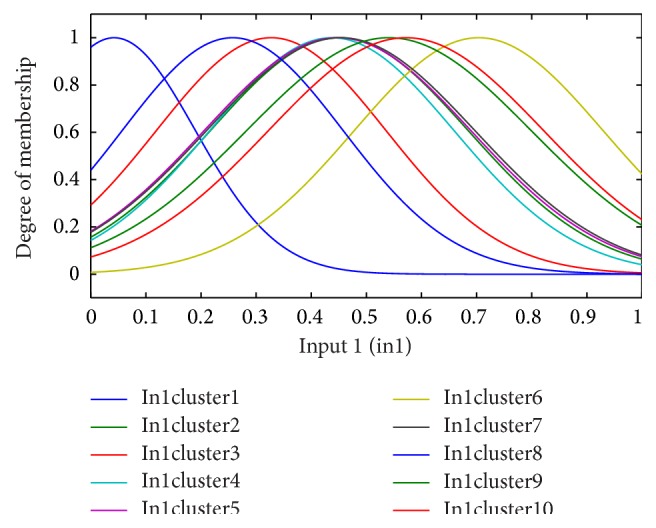
The fuzzy functions selected for input 1, underlying disease (diabetes), for the 6-month period.

**Figure 4 fig4:**
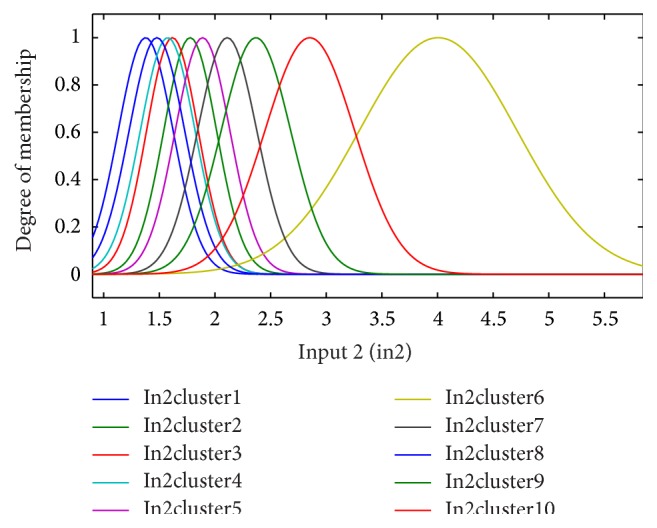
The fuzzy functions selected for input 2, diastolic blood pressure (dbp), for the 6-month period.

**Figure 5 fig5:**
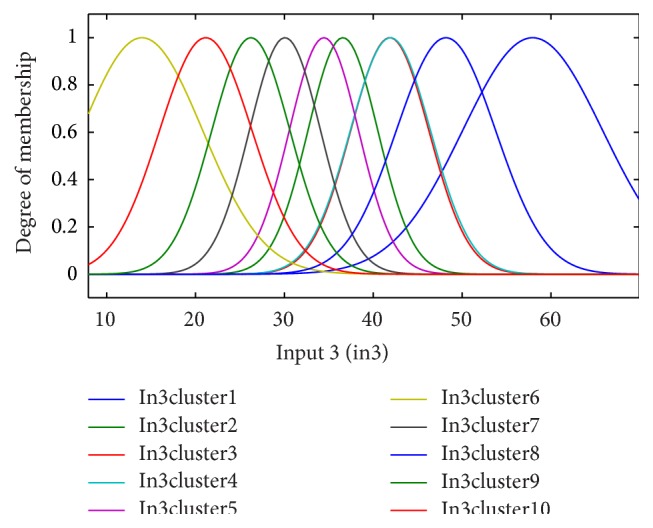
The fuzzy functions selected for input 3, weight, for the 6-month period.

According to the clinical dataset recorded from patients, fuzzy clustering of the four significant input variables is shown for the period of 6 months in Figures 3–6. Inputs 1, 2, 3, and 4 were assigned to underlying disease (as diabetes), diastolic blood pressure (dbp), weight, and GFR, respectively, in the modeling and figures. For each input, 10 clusters were considered. According to the trial and error, 10 clusters provide better results (with the lowest error).

### 3.2. Training ANFIS

The rules in the rule base build a fuzzy inference system. After training, it was converted to a fuzzy inference system called ANFIS. ANFIS training was performed using MATLAB.

**Figure 7:**
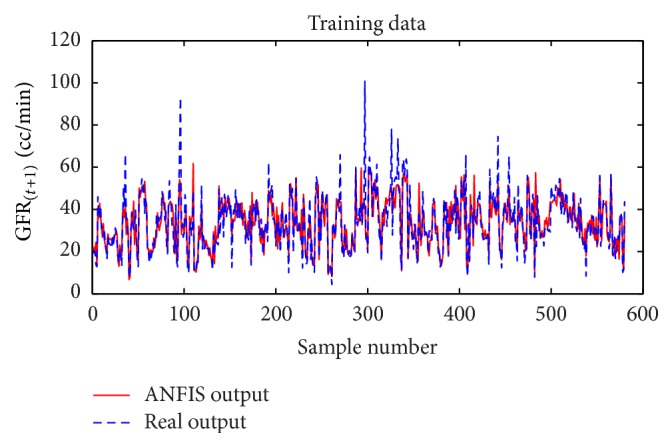
Comparison of the ANFIS prediction and real GFR_(*t*+1)_ values for the training dataset at 6-month interval.

**Figure 8:**
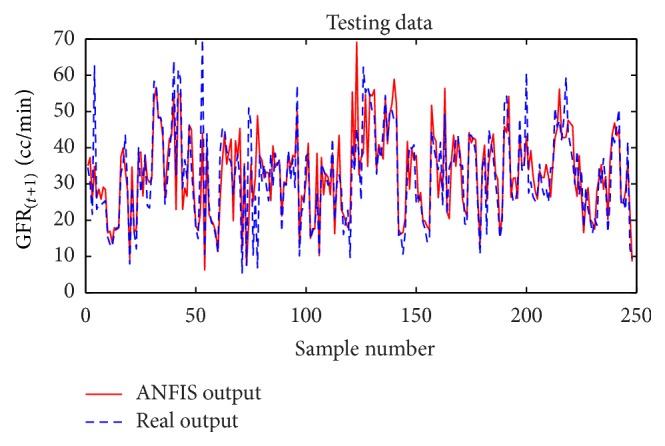
Comparison of the ANFIS prediction and real GFR_(*t*+1)_ values for the test dataset at 6-month interval.

Considering GFR modeling at one, two, or three future periods with the selected input variables including underlying disease, diastolic blood pressure, weight, and GFR_(*t*)_ values, the number of observations for modeling was 674 rows. 30% of the data, 202 records, for the test dataset, and 472 records were used as training dataset to train the ANFIS. The trained ANFIS was used for estimating GFR at subsequent 6, 12, or 18 months. Figures 7 and 8, respectively, show the performance of ANFIS model for training and test datasets for the 6-month period. The GFR changes are well followed by ANFIS in test dataset (Figure 8).

**Figure 9:**
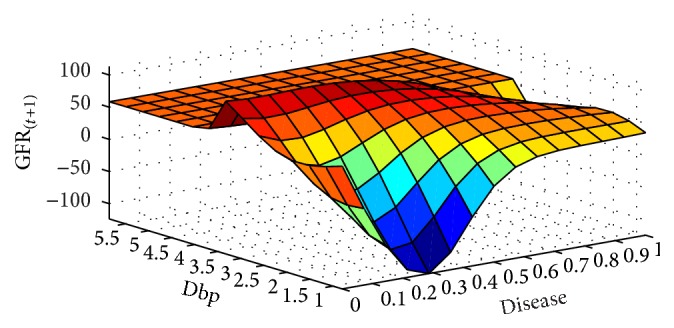
GFR_(*t*+1)_ function and its relationship with the underlying disease and dbp variables for the 6-month period.

**Figure 10 fig10:**
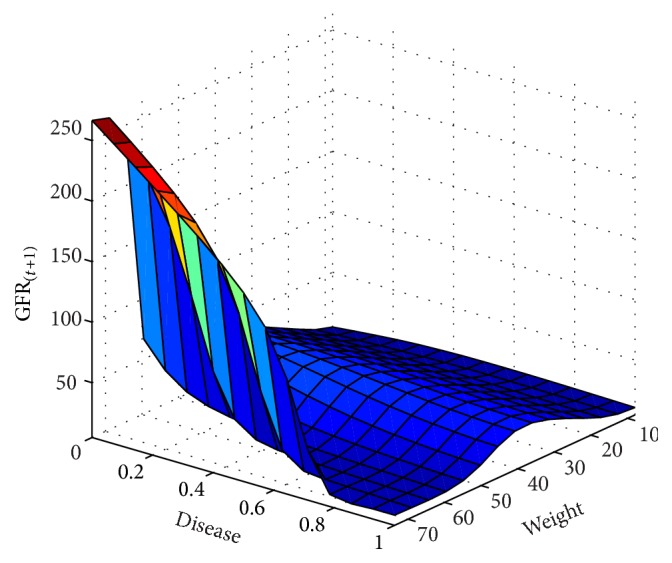
GFR_(*t*+1)_ function and its relationship with the underlying disease and wt variables for the 6-month interval.

**Figure 11 fig11:**
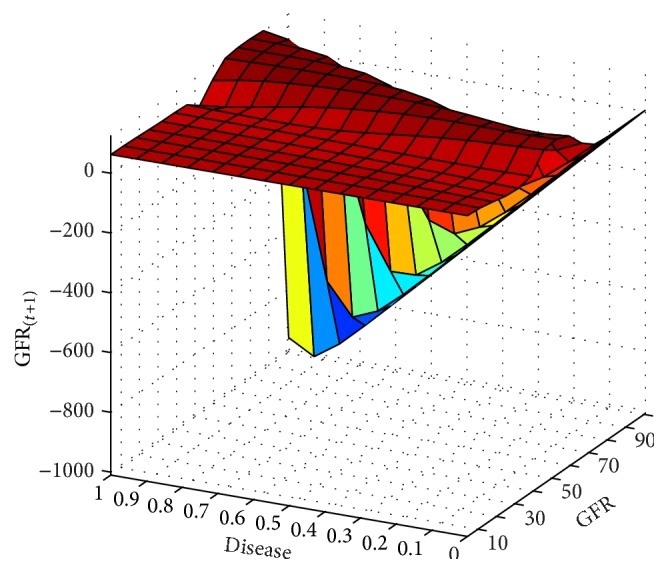
GFR_(*t*+1)_ function and its relationship with the underlying disease and gfr variables for the 6-month period.

**Figure 12 fig12:**
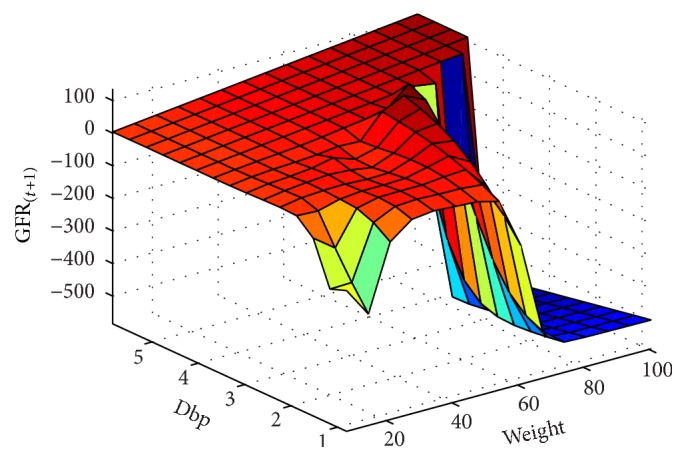
GFR_(*t*+1)_ function and its relationship with the dbp and wt variables for the 6-month period.

**Figure 13 fig13:**
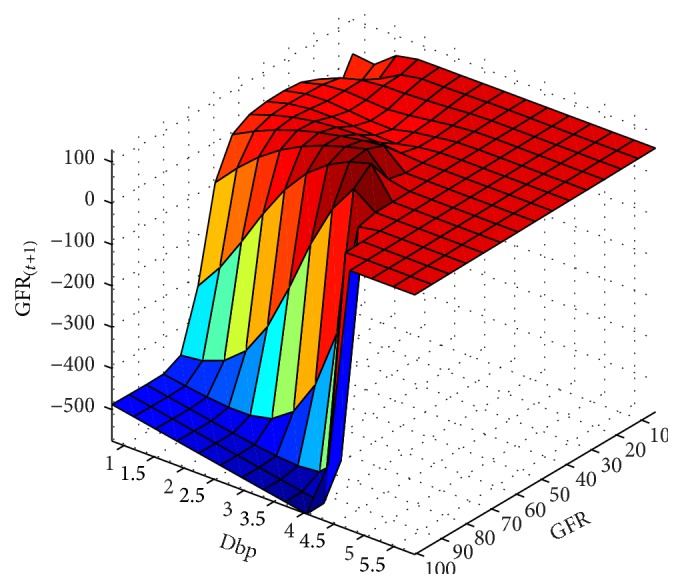
GFR_(*t*+1)_ function and its relationship with the dbp and gfr variables for the 6-month period.

**Figure 14:**
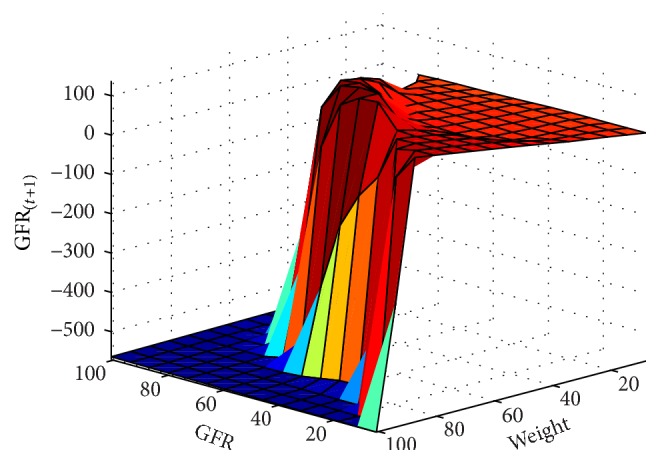
GFR_(*t*+1)_ function and its relationship with the wt and gfr variables for the 6-month period.

In next step, the GFR function was estimated for the 6-month period according to the patients' records. The results are presented as fitted surfaces for the output variable, GFR_(*t*+1)_, which is the value of GFR at 6-month interval (Figures 9 [Fig fig10] [Fig fig11] [Fig fig12] [Fig fig13]–14). The fitted surfaces also show the relationship between input variables and GFR.

**Figure 15:**
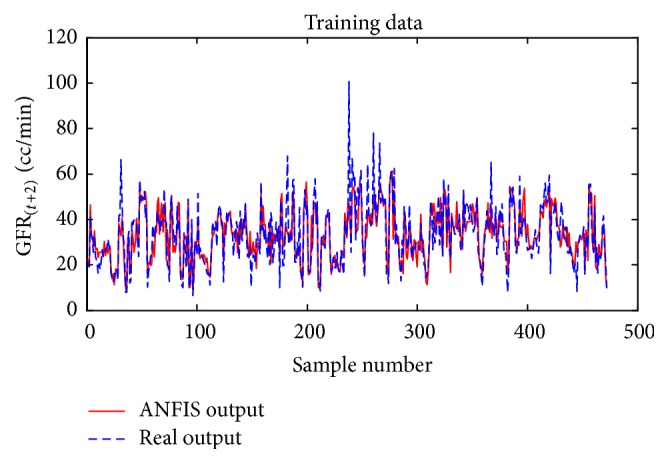
Comparison of the ANFIS prediction and real GFR_(*t*+2)_ for the training dataset for the 12-month period.

**Figure 16 fig16:**
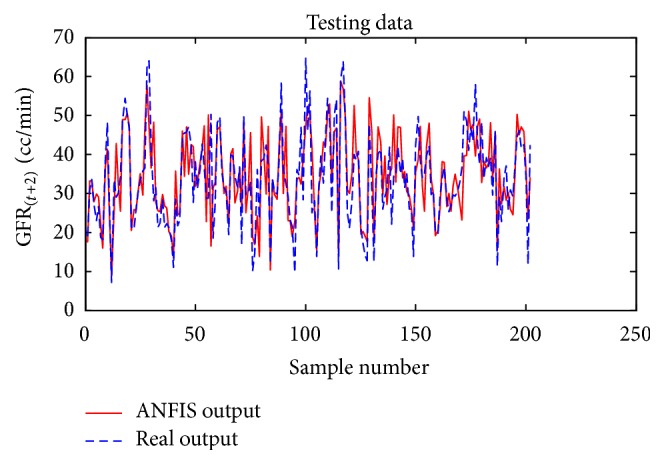
Comparison of the ANFIS prediction and real GFR_(*t*+2)_ values for the test dataset for the 12-month period.

**Figure 17 fig17:**
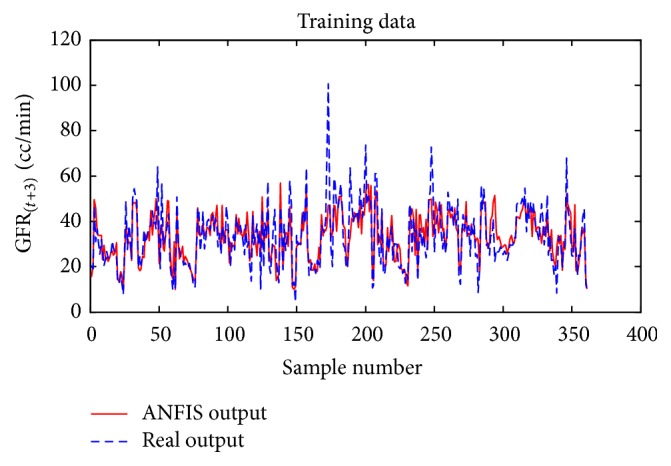
Comparison of the ANFIS prediction and real GFR_(*t*+3)_ values for the training dataset for the 18-month period.

**Figure 18:**
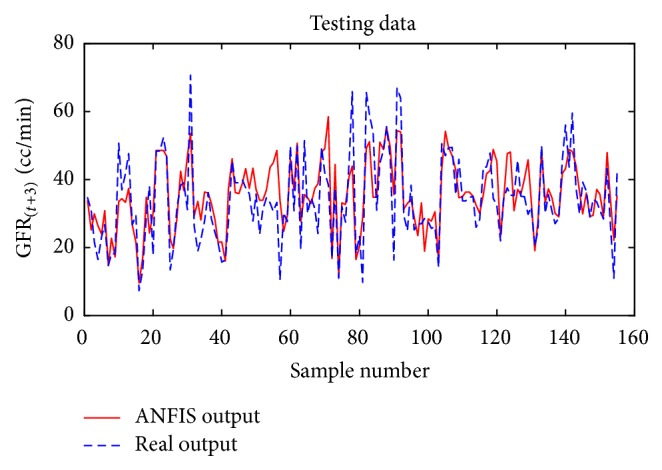
Comparison of the ANFIS prediction and real GFR_(*t*+3)_ values for the test dataset for the 18-month period.

In next step, the model was used to predict the GFR values at sequential 12- and 18-month intervals (Figures 15 [Fig fig16] [Fig fig17]–18). The results showed that ANFIS was able to predict GFR with an acceptable accuracy for the test data. The assessments showed that, despite increasing the forecast period, ANFIS was still able to predict GFR with an acceptable accuracy (Figures 15–18).

For further assessments and comparisons of the modeling, the results were examined based on the error criteria. Considering the variations of the results, appropriate error criteria should be used to evaluate the accuracy and efficiency of the predicting model. Three criteria were selected to evaluate and compare the accuracy of the fuzzy model: Mean Square Error (MSE), Mean Absolute Error (MAE), and Normalized MSE (NMSE). Of them, the NMSE is preferred since it provides the normalized error ranged from 0 to 100 percent. The formulas for error criteria are expressed by (1) as follows:

(1) MSE=∑i=1Nyi−y^i2N,MAE=∑i=1Nyi−y^iN,NMSE=∑i=1Nyi−y^i2∑i=1Nyi2×100.

Table 3 compares the ANFIS results at 6-, 12-, and 18-month intervals for the training and test datasets based on the error criteria. The comparisons show the high accuracy of ANFIS model for predicting GFR values for all sequential 6-, 12- and 18-month intervals in both training and test datasets. With reference to the low error rate of test data, the proposed ANFIS could be generalized to predict GFR level in new patients.

As the number of input variables increases, the modeling error decreases. As the ratio of observations to variables increases, the reliability increases, and thereby estimation error of the model parameters decreases. The modeler prefers fewer variables, provided that the model error remains low. Reviewing the relationships between the independent and dependent variables, among 10 independent variables, four of the most important independent variables with the highest correlation with the dependent variable (GFR) were selected. The accuracy of models was enhanced through eliminating other variables.

The present study was conducted in specific clinical situations (predicting the CKD progression) by integrated fuzzy modeling. This can be helpful in expediting medical applications. However, a question whether the numbers of time periods or lags considered for prediction affect modeling and error rate arises. It is noteworthy that a modeler always prefers to do forecasts for long periods, provided that the forecast error is not very high. Therefore, the time delay (*p*) should be selected so that is a tradeoff between time interval and the modeling error. The implementation of models and comparisons confirm that ANFIS provides highly reliable results for the all 6-, 12- and 18-month periods. Therefore, the ANFIS is considered as an acceptable model. The main reasons for preferring the ANFIS are as follows.

- The model provides the ability to model fuzzy variables. In this regard, the uncertainty can be appropriately modeled.
- The model is able to estimate and predict GFR, so that the error rate has been reduced to 4% in some cases.
- In addition to GFR prediction, the model produces a fuzzy database. The database shows the complex relationships between experimental inputs and GFR as simple linear models in different modeling environments. The transparency of GFR membership function in the ANFIS is the advantage of ANFIS compared to other models such as statistical model like regression or neural network like multilayer perceptron neural network.

## 4. Perspectives of Practical Implementation

The proposed model can be used as a core computational component in a perspective medical decision support system (MDSS) to help physicians in making appropriate decisions about the time of renal replacement therapy. This system can take physiological parameters as input and predict the GFR values at future intervals. Using appropriate threshold value of GFR, nephrologists can effectively manage the CKD patients. To build such MDSS, it is necessary to complement the predicting model through evaluating the other possible influential parameters in kidney function.

## 5. Discussion

The results of modeling and forecasting by ANFIS networks show that the models have a reliable accuracy for all periods of 6, 12, and 18 months. Therefore, fuzzy model could be used to predict GFR with a high reliability. The main challenge in modeling the renal failure progression is the high uncertainties of the human body as the environment as well as high dynamicity of the disease. Statistical and machine learning based prediction models cannot effectively overcome these problems. However, the proposed model could significantly control these issues using neurofuzzy approach. Furthermore, our model can accurately predict the GFR values in long future period so that increasing the forecasting period to 12 and 18 months do not reduce the accuracy of the prediction model (4.88% NMAE). This model can be used in clinical practice.

One of the main concerns of clinicians has been the efficacy of CKD patients' supervision and regular follow-up on decreasing the speed of disease progression and prevention of its inevitable complications. Therefore, the use of efficient predicting model proposed in this study for decision support system in the field of kidney diseases as well as CKD management in a more quantitative manner may be an important strategy for reducing its burden. Using this model, it is possible to monitor the impact of each variable, routinely measured in CKD patients. Rucci et al. found that patients with proteinuria and a baseline estimated GFR (eGFR) of >33 mL/min/1.73 m^2^ had faster decline of GFR (2.8 mL/min/1.73 m^2^) than those with baseline eGFR of <33 mL/min/1.73 m^2^ and a baseline serum phosphorus of >4.3 mg/dL in their retrospective study. Among patients without proteinuria, those younger than 67 years exhibited a significantly faster progression, which was even faster for the subgroup with diabetes. Among patients aged older than 67 years, females had more steady eGFR than men [12].

Our results in comparison with the results of Tian et al. [23] on the survival time of hemodialysis patients as well as other studies using machine learning based methods are of high validity and reliability. Furthermore, choosing appropriate input variables is the most important feature of the model that can improve prediction accuracy. One of the limitations of this study may be the presence of intrinsic errors in eGFR calculated with MDRD. Recently, Fan et al. introduced a new method for eGFR calculation based on cystatin C and concluded that it may be a better filtration marker than the equations based on serum creatinine value particularly in elderly patients [24]. To validate our proposed model, we can model the renal failure worsening framework using different equations of eGFR.

## 6. Conclusion

An ANFIS based model was developed for modeling the renal failure progression and predicting the renal failure time. The model could accurately (>95%) predict the GFR for sequential 6-, 12-, and 18-month intervals. The main limitation of this study from the clinical point of view was that urine protein was not among the variables evaluated for the prediction of GFR in 6 to 18 months.

## Figures and Tables

**Figure 6:**
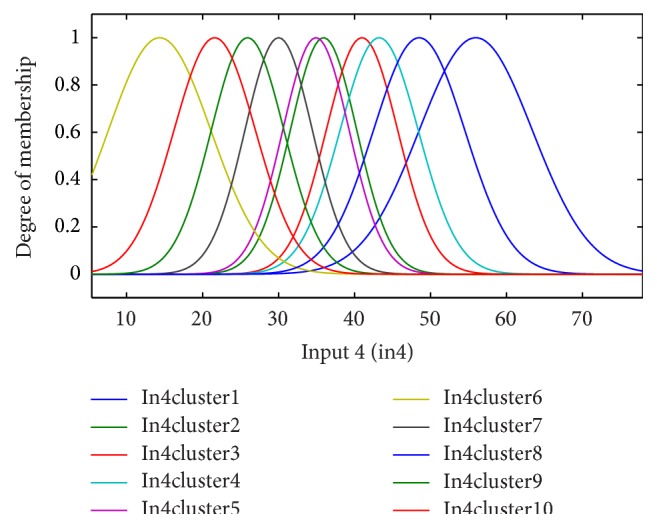
The fuzzy functions selected for input 4, GFR_(*t*)_, for the 6-month period.

**Table 1:** Baseline characteristics of the study population (*n* = 465).

Variable	Mean ± standard deviation
GFR (cc/min/m^2^)	34.8 ± 11.0
Age (years)	61.3 ± 14.9
BMI (kg/M^2^)	26.5 ± 4.2
Systolic blood pressure (CmHg)	14.0 ± 2.7
Diastolic blood pressure (CmHg)	7.8 ± 1.3
Hemoglobin (gr/dL)	12.6 ± 2.1
Phosphorus (mg/dL)	4.10 ± 0.8
Uric acid (mg/dL)	8.1 ± 1.9
Cholesterol (mg/dL)	198.5 ± 58.9
Triglyceride (mg/dL)	205.6 ± 134.5

**Table 2:** The correlation coefficients between all inputs and target output (GFR_*t*+*p*_) for 6-month period. dbp = diastolic blood pressure; Cr: creatinine; Ca: calcium; P: phosphorus; underlying disease (Diabetes); GFR: glomerular filtration rate.

Input	Input number	Correlation coefficient between input *i* and output
Underlying disease_(*t*)_	1	0.2505
Sex_(*t*)_	2	0.0706
Age_(*t*)_	3	−0.1043
dbp_(*t*)_	4	0.7145
Cr_(*t*)_	5	0.0322
Ca_(*t*)_	6	−0.2224
P_(*t*)_	7	−0.1444
Uric acid_(*t*)_	8	0.1089
Weight_(*t*)_	9	0.8120
GFR_(*t*)_	10	0.5196

**Table 3:** Comparison of error criteria for the training/test datasets for 6-, 12-, and 18-month periods. MSE: Mean Square Error; MAE: Mean Absolute Error; NMSE: Normalized MSE.

		Training dataset ANFIS	Test dataset ANFIS
6 months	MSE	48.7053	58.6253
	MAE	4.9960	4.7654
	NMSE	3.7428%	4.7676%

12 months	MSE	53.1676	54.885
	MAE	5.1170	5.5010
	NMSE	4.1714%	4.3019%

18 months	MSE	62.5255	64.0022
	MAE	5.5640	5.9302
	NMSE	4.8709%	4.8787%
